# Beyond the joints: mechanistic insights and multidisciplinary strategies for spondyloarthritis-associated uveitis

**DOI:** 10.3389/fimmu.2025.1715107

**Published:** 2025-12-03

**Authors:** Shuyue Pan, Ling Xiang, Zhi Cao, Zhi Yang, Rui-Juan Cheng

**Affiliations:** 1 Department of Rheumatology, Fifth People’s Hospital Affiliated to Chengdu University of Traditional Chinese Medicine, Chengdu, China; 2 Department of Radiology, Fifth People’s Hospital Affiliated to Chengdu University of Traditional Chinese Medicine, Chengdu, China; 3 Department of Rheumatology and Immunology, West China Hospital, Sichuan University, Chengdu, China

**Keywords:** spondyloarthritis, uveitis, HLA-B27, extra articular manifestations, biologics

## Abstract

Spondyloarthritis (SpA) is a group of diseases characterized by chronic inflammation and extra-articular involvement of tissues and organs. Acute anterior uveitis (AAU), the most common extra-articular manifestation of SpA, typically presents as a unilateral acute disruption of the blood-aqueous barrier, characterized by photophobia, redness of the eye, pain, and blurred vision. The underlying pathogenesis is not fully understood but is thought to involve genetic factors, gut microbiota, and dysregulation of the immune system. Corticosteroids remain the cornerstone of AAU treatment. However, alternative therapies such as nonsteroidal anti-inflammatory drugs (NSAIDs), disease modifying antirheumatic drugs (DMARDs), and biologics have also demonstrated utility. In recent years, biologics have gained increasing attention due to their efficacy and safety. This review summarizes recent research findings and advancements in the diagnosis and management of spondyloarthritis-associated uveitis.

## 1 Introduction

Spondyloarthritis (SpA) is a group of systemic chronic inflammatory rheumatic immune diseases characterized by significant clinical heterogeneity. The global prevalence of SpA varies significantly, ranging from 0.20% in Southeast Asia to 1.61% in the Arctic regions (1). Common subtypes include ankylosing spondylitis (AS), psoriatic arthritis (PsA), reactive arthritis (ReA), inflammatory bowel disease-associated arthritis (IBD-A), and undifferentiated spondyloarthritis (uSpA) (2). These conditions typically affect sacroiliac joints, spine, and peripheral joints, causing inflammatory low back pain and/or asymmetric peripheral arthritis. They are often accompanied by extra-articular multisystem involvement (3). Ocular involvement is a common extra-articular manifestation in SpA patients, with uveitis being the most common type (4). Early recognition, diagnosis and systematic treatment of the SpA complicated by uveitis are critical for improving patient outcomes. This article reviews the clinical manifestations, etiology, pathogenesis, and treatment of SpA-associated uveitis.

## 2 Methods

### 2.1 Search strategy

In this review, a systematic search strategy was employed to identify relevant peer-reviewed articles from 2020 to 2025 in the electronic databases of PubMed, Embase, China National Knowledge Infrastructure (CNKI), and Wanfang, without any language restrictions applied. The search terms comprised "Spondyloarthritis", "Uveitis", "Ankylosing Spondylitis", "Psoriatic Arthritis" ,"Enteropathic Arthritis", "Targeted Therapy" ,"HLA-B27", and "Spondylitis". Additionally, the reference lists of retrieved articles were examined to identify other relevant studies for inclusion.

### 2.2 Inclusion criteria and exclusion criteria

Inclusion Criteria: (1) Adult patients (age ≥18 years) with a definitive diagnosis of spondyloarthritis (including axial SpA, ankylosing spondylitis, reactive arthritis, psoriatic arthritis, or enteropathic arthritis) accompanied by uveitis of any type (e.g., anterior, intermediate, posterior, or panuveitis), regardless of race or sex. (2) Studies investigating any aspect of SpA-associated uveitis, including but not limited to epidemiology, pathogenesis, clinical manifestations, diagnosis, efficacy and safety of treatments.

Exclusion Criteria: (1) Pediatric patients (age <18 years). (2) Patients with uveitis secondary to other definite causes (e.g., infection, trauma, or other autoimmune diseases such as sarcoidosis or Behçet’s disease). (3) Case reports (with fewer than 2 cases) and studies from which data could not be extracted.

All retrieved records were imported into a reference management software (e.g., EndNote) for automatic removal of duplicates. Two authors (S-YP and LX) conducted the electronic search independently, and any discrepancies were resolved by a third author (ZC).

## 3 Clinical manifestations and characteristics

Extra-articular manifestations of SpA are often overlooked and primarily involve multiple systems, including eyes, skin, gastrointestinal tract, bone, heart, lung and kidney (5). Among these, uveitis is regarded as one of the most prevalent extra-articular manifestations of SpA, with acute anterior uveitis (AAU) representing the predominant form, accounting for approximately 60% to 90% of cases (5). The prevalence of AAU varies in different regions, with higher rates observed in in Europe and North America compared to Asia (6). In a study by Rupali et al. (7), 225 SpA patients were evaluated and 42 patients (18.7%) were found to have uveitis. Uveitis was more common in males than in females, with ratio of 2.5:1. Furthermore, the incidence of uveitis increases with the duration of SpA. At the time of diagnosis, the incidence is approximately 10.5%, rising to 46.6% after 30 years of disease (8). The prevalence of uveitis also varies among different subtypes of SpA: AS is reported to have the highest prevalence (32.2%), followed by IBD-A at 36.9% and PsA at 25.1% (9).

Uveitis refers to the inflammation of the uveal tract of the eye, which includes the iris, ciliary body and choroid. It can be classified based on the anatomical location of the inflammation into anterior, intermediate, posterior, or panuveitis (involving all three segments) (10). In Western countries, anterior uveitis is the most common form, while in developing countries, posterior uveitis and panuveitis are more frequently observed (11). Clinically, uveitis is further categorized into acute (lasting less than 3 months), chronic (more than 3 months), or recurrent uveitis (recurring after resolution from a prior episode) (12). SpA-associated uveitis most commonly presents as AAU. When uveitis persists, severe disruption of the blood-aqueous barrier can occur and lead to significant infiltration of inflammatory cells in the anterior chamber. This process is usually accompanied by fibrin exudate and hypopyon, resulting in adhesion between the iris and lens (13). In addition, severe and/or recurrent AAU may lead to structural complications, such as cataract, elevated intraocular pressure, glaucoma, and cystoid macular edema, which can progress to chronic anterior uveitis (14, 15).

AAU primarily affects the anterior segment structures of the eye, including the iris and ciliary body, but it can also involve the posterior segment through the dissemination of immune mediators. SpA-associated uveitis is typically non-granulomatous, acute, and unilateral (16). On the one hand, the prognosis for AS-related uveitis is generally good, with most cases resolving spontaneously within 4 to 6 weeks, although recurrence is common. Patients with AS-related uveitis are often young adults, and while the majority experience some degree of visual impairment, severe vision loss is rare (17). In the early stages of the disease, ciliary muscle spasm can cause photophobia and localized ocular discomfort. As the disease progresses, patient may develop characteristic symptoms such as photophobia, red eye, lacrimation, pain, and blurred vision. On the other hand, uveitis associated with IBD-A and PsA more commonly affect both eyes. It tends to have a more insidious onset, slow progression and prolonged duration. They may present as vitritis, affecting the middle and posterior segments of the eye, and can lead to various severe ocular complications (18, 19).

**Figure 1:**
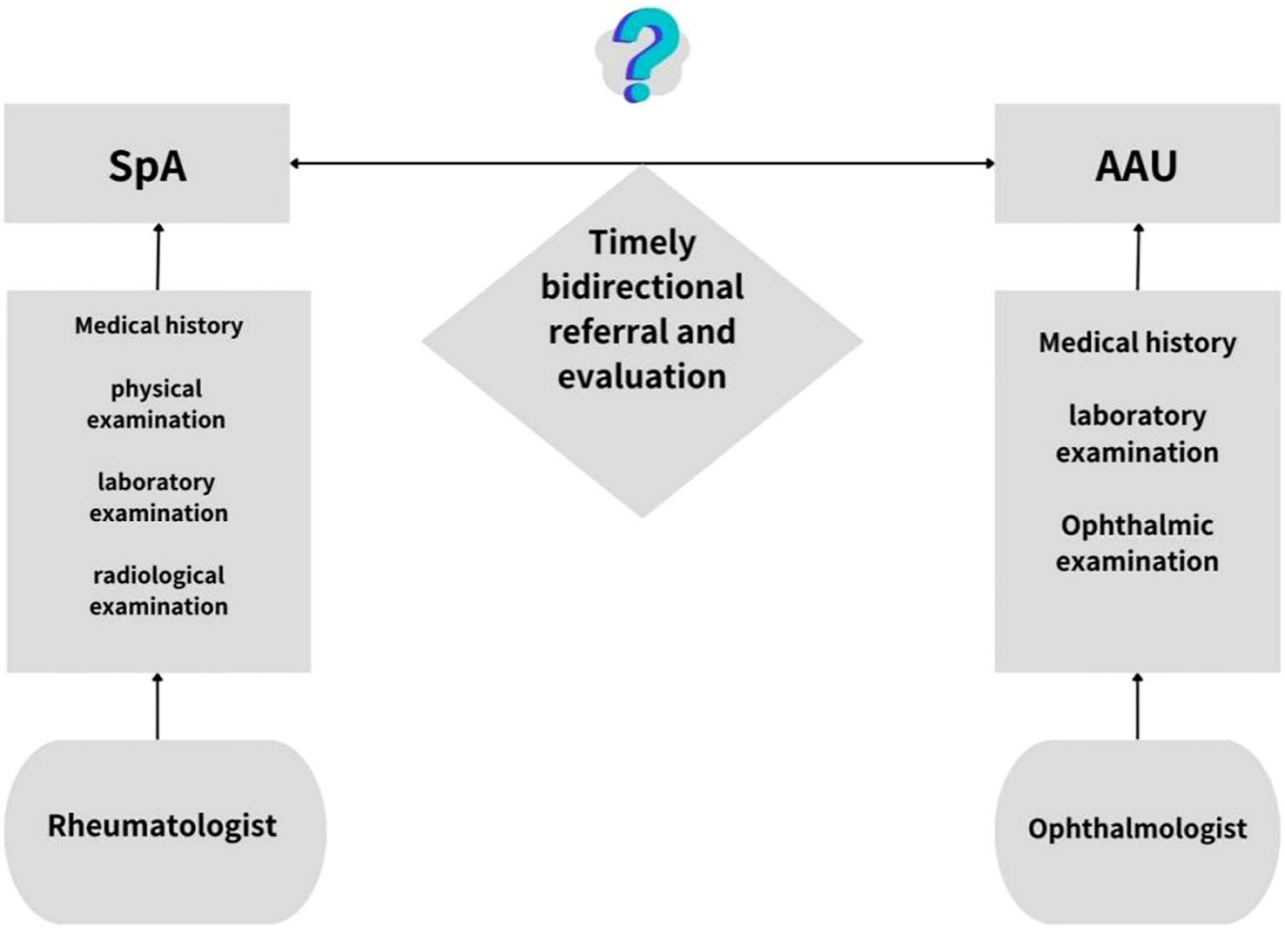
Diagnostic flowchart for AAU in spondyloarthritis.

Risk factors associated with secondary uveitis in patients with SpA include age, smoking history, delayed diagnosis, long disease duration, HLA-B27 positivity, family history of SpA, and radiographic damage (20). These factors provide valuable guidance for clinical practice. For those patients with SpA risk factors, such as arthritis and family history, ophthalmologists and rheumatologists should maintain a high level of vigilance and timely carry out the necessary examinations and evaluations (**Figure 1**). Uveitis associated with SpA is generally a form of non-infectious inflammation. During an acute episode of SpA-associated AAU, intraocular pressure in the affected eye is typically lower than that of the contralateral eye due to the release of prostaglandins. This characteristic can help differentiate SpA-associated AAU from viral anterior uveitis and other types of uveitis (21). The prognosis of SpA-associated uveitis largely depends on the timeliness of diagnosis and treatment. If diagnosed and treated early, most patients experience a favorable outcome. Therefore, detailed eye examination and systematic evaluation are particularly important (22). Eye examinations mainly include vision assessment, intraocular pressure measurement, slit lamp examination, optical coherence tomography (OCT), and fundus examination. Slit lamp examination provides high magnification images of the eye, enabling observation of the distribution of inflammatory cells, fibrin exudates, and provide overall evaluation of intraocular inflammation. OCT is a valuable monitoring tool that can identify macular edema early and assist in management.

Diagnosis of SpA-associated uveitis should be targeted based on thorough review of medical history, symptoms, uveitis course, ocular signs, and laboratory data (23). The Standardization of Uveitis Nomenclature Working Group has released the main classification criteria for SpA/HLA-B27-related AAU (24). The first step in classifying is to exclude infectious causes of uveitis. Then the following situations can be classified as SpA/HLA-B27-AU: For patients presenting with acute or recurrent acute, unilateral or unilateral alternating AAU, accompanied by spinal arthritis or positive HLA-B27 test; or patients with a history of recurrent acute, unilateral, or unilateral alternating in the context of a chronic disease, along with spinal arthritis or positive HLA-B27 test; or patients with AAU accompanied by spondyloarthritis and positive HLA-B27. In addition, the Dublin uveitis assessment tool (Duet) (25)recommends that AAU patients who present with inflammatory back pain or HLA-B27 positivity, regardless of the presence of axial or peripheral SpA features, should be referred to a rheumatology specialist for a comprehensive evaluation. Such interdisciplinary collaboration is crucial for the early screening, identification, and treatment of potential SpA cases (26).

## 4 Research progress on etiology and pathogenesis

The exact etiology and pathogenesis of SpA-associated uveitis remain complex and are not completely understood. Current research shows that it may be related to genetic factors, gut microbiota, infection factors and dysregulation of immune system (**Table 1**). We summarized the research in understanding the mechanism underlying AAU.

**Table 1:** Overview of the pathological mechanisms of spondyloarthritis-associated uveitis.

Pathogenesis	Key points	Correlation
Genetic factors	Arthritis/uveal peptide hypothesis HLA-B27 misfolding hypothesis Innate immune recognition of abnormal HLA-B27 Genetic association of AAU and SPA	Presentation of abnormal peptides Autoimmune response Endoplasmic reticulum stress The activation of the unfolded protein response Expression and recognition of molecular abnormal forms HLA-B27/HLA-DR13/ERAP/etc
Gut microbiota	Bacterial enrichment and dysbacteriosis	Intestinal permeability changes and immune system activation
Infectious factors	Genitourinary and intestinal infections Microbial antigens and antibodies	Triggering of corresponding microbial infection Molecular mimicry
Dysregulation of immune system	Abnormal activation of immune cells Dysregulation of cytokine networks Abnormal activation of signaling pathways	T cells, NK cells, macrophages, dendritic cells, B cells, etc TNF-α、IL-17 and IL-23 Wnt, NF-kB, Jak-STAT and other signaling pathways

### 4.1 Genetic factors

Genetic factors play a significant role in SpA, with the strongest association being with the human leukocyte antigen HLA-B27. Approximately 50% of AAU patients are HLA-B27 positive (27). HLA-B27 increases the risk of AAU in patients with SpA, and for HLA-B27 positive individuals without SpA, the risk of developing AAU is 23 times higher than that of the general population (28). Studies have shown that as the duration of SpA increases, the incidence of uveitis also rises, particularly among HLA-B27 positive patients, with an odds ratio of 4.2 (29). This highlights the importance of routine HLA-B27 testing in patients with AAU, as it may influence disease severity and prognosis. A retrospective study conducted in Sydney further supported routine testing, showing that HLA-B27 positive patients had relatively poorer visual outcomes after review of 241 AAU cases (30). Currently, there are three main hypotheses explain the role of HLA-B27 in the pathogenesis of SpA-associated uveitis: Arthritis/uveal peptide hypothesis, HLA-B27 misfolding hypothesis and innate immune recognition of abnormal HLA-B27 (31).

According to the Arthritis/uveal peptide hypothesis, HLA-B27, as a major histocompatibility complex (MHC) class I molecule, presents processed self-peptides or antigens to the surface of cytotoxic CD8+T cells. Upon recognition, this process can lead to auto-reactivity, inducing inflammation in the joints or eyes, resulting in arthritis and uveitis (5). The fundamental mechanism of this process revolves around the recognition of HLA-B27 bound self-peptide complexes by the T-cell receptor (TCR), which subsequently triggers a specific autoimmune response. Hermann et al. (32)tested the synovial fluid of four patients with ReA and two patients with AS. From these samples, they obtained 354 αβ-TCR CD8+T lymphocyte clones (TLCs). Autoreactive cytotoxic T cells were identified in five of the six patients, with five demonstrating HLA-B27 restricted killing of uninfected cell lines. Yang et al. (33) identified a clonally expanded TCR (TRBV9/TRAV21) associated with AS and AAU. They propose that this TCR interacts with a common HLA-B27-peptide complex, thereby contributing to the pathogenesis of both conditions. To date, over 200 HLA-B27 subtypes have been reported based on nucleotide sequence polymorphisms. Among these, HLA-B27*04 and HLA-B27*05 subtypes are associated with SpA and AAU, while HLA-B27*09 shows no association (34, 35). A comprehensive analysis of the eight most common HLA-B27 subtypes revealed considerable overlap among the peptidomes, suggesting that it may be the quantity rather than the quality of peptides that contribute to disease occurrence – a discovery of considerable significance in understanding the pathogenesis of SpA-associated uveitis (36). Despite advancements in comprehending HLA-B27 antigen presentation, critical questions regarding the specific self-peptides involved and the mechanisms of immune activation remain unanswered, necessitating further investigation.

The HLA-B27 misfolding hypothesis posits that the inherent instability of the HLA-B27 molecule makes it prone to misfolding. This can trigger endoplasmic reticulum (ER) stress, thereby activating the unfolded protein response (UPR) (37). In some cases, the upregulation of UPR genes can further lead to the activation of the NF-κB signaling pathway, resulting in the production of pro-inflammatory cytokines such as TNF-α, IL-1, and IL-6, causing inflammation (38). It has also been reported that UPR may participate in the development of the disease by activating the IL-23/IL-17 axis (39). These pathways could all contribute to the development of SpA-associated uveitis.

In SpA-associated uveitis, the HLA-B27 molecule may directly participate in immune activation by forming dimers or free heavy chains (FHCs). The formation of dimers, which occurs in the absence of β2-microglobulin (β2m), is dependent on the Cys67 residue on its molecule (40). These “abnormal” cell surface forms can be recognized by the innate immune system, including natural killer (NK) cells or the killer cell immunoglobulin-like receptors KIR3DL1, KIR3DL2, and the immunoglobulin-like transcript 4 (ILT4) on T helper cells (41), thereby stimulating CD4+T lymphocytes to produce IL-17, initiating an immune response (42, 43). In a study observing KIR gene polymorphisms, HLA-B27 positive AS patients had higher levels of KIR2DL1 and KIR2DL5 compared to those without AS (44). Furthermore, research conducted by Vendelbosch et al. (45)indicated that the severity of AS in patients appears to be influenced by KIR genotypes. However, there were no significant differences between patients and controls in terms of KIR haplotypes, genes, or allele distributions.

In recent years, genome-wide association studies (GWAS) have identified numerous genetic links associated with AAU and SpA. Apart from HLA-B27, HLA-DR13 is also closely related to uveitis in PsA patients (46). Researchers have discovered that genetic variations in non-HLA regions may also be associated with SpA-associated uveitis. These including ERAP1, intergenic region 2p15, IL23R, IL-10-19, and IL-18R1-IL-1R1 genes, which predispose to uveitis and inflammatory bowel disease. In contrast genes such as IL-6R, KIF21B, and EYS appear to specifically predispose individuals to AAU (47). Furthermore, associations have also been found at previously unrecognized loci for AS and AU, including MERTK, KIFAP3, CLCN7, ACAA2 (48). Among these genes, the endoplasmic reticulum aminopeptidases ERAP1 and ERAP2 have genetic associations with SpA that are second only to HLA-B27. These enzymes regulate the length of peptides by trimming N-terminal amino acids, enabling the peptides to fit with MHC class I molecules and present them on the cell surface to CD8+ T cells or NK cells (49).

### 4.2 Gut microbiota

The human body, gut microbiota, host organism, and environment are interdependent mutually influence one another. Gut microbes are thought to play a crucial role in the pathogenesis of SpA-associated uveitis, but the exact mechanisms are not yet clear.

The diversity and composition of gut microbiota vary in different diseases. Studies have found that patients with AS have increased abundance of Lachnospiraceae, Ruminococcaceae, Rikenellaceae, Porphyromonadaceae, and Bacteroidaceae in the terminal ileum, while Veillonellaceae and Prevotellaceae are reduced (50).The Firmicutes/Bacteroidetes (F/B) ratio is critical for maintaining normal gut homeostasis, and an increase in Firmicutes species abundance has been linked to AS (51). Compared with healthy controls, uveitis patients showed enrichment of Prevotella and Streptococcus, and decreased diversity of Faecalibacterium, Bacteroides, Lachnospira, Ruminococcus, Lachnospiraceae and Ruminococcaceae (52). Recently, studies have shown that patients with AAU and patients with SpA and Crohn’s disease have increased Collinsella and decreased Lachnospiraceae, Blautia and Fusicatenibacter (53). However, it remains unclear whether dysbiosis is a cause or consequence of inflammation, and methodological variations limit the comparability and generalizability of microbiota findings.

Animal models provide a valuable tool for investigating the role of gut microbiota in the pathogenesis of SpA-associated uveitis. Experiments have shown that HLA-B27 transgenic rats raised in a sterile environment have a reduced incidence of intestinal and joint inflammatory diseases, confirming the significant role of microbiota in disease development (54). Oral antibiotic treatment can significantly reduce ocular inflammation compared to intraperitoneal administration, suggesting that the modulation of gut microbiota has a direct impact on ocular inflammation (55). Furthermore, a retrospective study from Taiwan has demonstrated a clear association between the progression of SpA and the severity of intestinal inflammation, further underscoring the interconnectedness of gut and systemic immune responses (56).

Under normal conditions, the gut microbiota maintains the integrity of the mucosa and supports the homeostasis of the immune system. However, dysbiosis may alter the intestinal permeability, allowing bacterial products or metabolites to cross the intestinal barrier and enter the bloodstream and tissues, triggering immune responses and inflammation (57). This is also associated with an elevation in the levels of intestinal inflammation markers, such as calprotectin (58). Medications aimed at reducing intestinal permeability may therefore help decrease the occurrence of uveitis. Additionally, dysbiosis of the gut microbiota can activate Paneth cells (PCs), causing release of pro-inflammatory cytokines IL-23 and IL-7, thereby inducing the differentiation and aggregation of type 3 innate lymphoid cells (ILC3) and mucosal-associated invariant T cells (MAIT) (51). This process results in increased levels of IL-17A and IL-22, further compromising the intestinal barrier and perpetuating inflammation (59).

### 4.3 Infectious factors

In recent years, it has become increasingly evident that infectious factors play a significant role in immune-mediated diseases. Infections of the genitourinary system (e.g. Chlamydia trachomatis) and gastrointestinal system (e.g. Campylobacter, Salmonella, Shigella, Yersinia) can lead to development of ReA (60). In patients with ReA, the presence of microbial antigens in the synovial fluid can be detected, and these patients exhibit a positive clinical response to antibiotic therapy (61), indicating that infectious agents play a direct role in disease onset. Further research has found that the serum levels of antibodies against Gram-negative bacteria are elevated in patients with HLA-B27 positive AAU and SpA, providing additional evidence of microbial involvement in these conditions (62). Helicobacter pylori has also been identified as a potential pathogen associated with the pathogenesis of AAU and SPA (62), although its exact mechanism of action remains unclear. These findings provide strong evidence for the role of microbial triggers in the pathogenesis of AAU and other immune-mediated diseases.

Infectious factors may interact with individual genetic susceptibility to jointly promote the occurrence of disease. Specifically, bacterial antigens can cross-react with self-antigens through molecular simulation to trigger autoimmune reactions (63). This mimicry enables the polypeptides of some microorganisms to resemble the structure of self-antigens, thereby reacting with host immune cells. This mechanism was further supported by a study conducted by bodis (64) and others, which demonstrated that peptides from various microorganisms can bind HLA-B27 molecules, thereby inducing immune responses in the eyes and joints, leading to local inflammation. However, microorganisms have not been detected in the aqueous humor of AAU patients, suggesting that direct microbial invasion of the eye is unlikely. Instead, studies propose that the presence of an unidentified microbiome may regulate the inflammatory response in the eye, contributing to disease pathogenesis (65).

### 4.4 Dysregulation of immune system

Uveitis is an intraocular inflammatory disease, and the dysregulation of immune system is an important factor in the development of AAU. The pathogenesis of uveitis involves a variety of immune cells and complex molecular mechanisms, including T cells, NK cells, macrophages and dendritic cells. IHLA-B27 plays a pivotal role in this process. It has been shown that HLA-B27 can form homodimers that present on the surface of T cells, monocytes and NK cells. These homodimers bind to killer cell immunoglobulin like receptor (KIR) which are displayed on peripheral blood mononuclear cells and synoviocytes of patients with SPA, thereby activating innate immunity (66). Specifically (67), the expression of killer cell Ig like receptor KIR3DL2 is increased in HLA-B27 AAU patients. This increased KIR3DL2 may stimulate CD4+T cell activation, leading to the secretion of a large number of pro-inflammatory cytokines. B cell infiltration can be observed in ocular tissues of patients with juvenile idiopathic arthritis associated uveitis, suggesting that B cells are also involved in the pathogenesis of uveitis (68). The activation of both the innate and adaptive immune systems leads to the release of various pro0inflammatory cytokines such as TNF-α, IL-17, and IL-23, which mediate the occurrence and progression of SpA through multiple signaling pathways including Wnt, NF-kB, and Jak-STAT (69, 70).

TNF is a 233-amino-acid protein that exists in two forms: transmembrane TNF (tmTNF) and soluble TNF (sTNF). After binding to the two receptors TNFR1 and TNFR2, it can activate multiple signaling pathways such as Wnt and NF-κB, initiating immune responses (71).In a prospective study, it was observed that the levels of TNF-α in the serum and aqueous humor of patients with uveitis were elevated, with a significantly higher concentration of TNF-α in the aqueous humor of HLA-B27 positive patients compared to HLA-B27 negative patients (72).

IL-17 is produced by various immune cells, including helper T cells (Th17), γδT cells, ILC3, and MAIT cells, with Th17 being the primary source (73). IL-17 can promote the recruitment and activation of neutrophils (74), increase angiogenesis and vascular permeability (75), thereby exacerbating the inflammatory response at the site of inflammation. Furthermore, it synergizes with TNF-α to release cytokines such as IL-1, TNF-α, and IL-6, forming a positive feedback loop that further amplifies the inflammatory response (76). This inflammatory cascade reaction may also be triggered by molecular simulations of invading microbial antigens and self-antigens (77).

Antigen presenting cells (APCs) such as dendritic cells (DCs), monocytes and macrophages produce IL-23. IL-23 is a key proinflammatory cytokine composed of P19 and P40 subunits (73). Sustained production of IL-17 is supported by IL-23, which promotes the proliferation and terminal differentiation of Th17 cells (78). Research indicates that the IL-23/IL-17 immunological axis plays a critical role in SpA-associated uveitis (79). Upon binding to cell surface or intracellular receptors of Th17 cells, γδT cells, ILC3, mast cells, and other cells, IL-23 activates the Jak2-STAT3 intracellular signaling pathway, inducing effector cells such as Th17 to produce pro-inflammatory cytokines like IL-17 and IL-22, while also disrupting the barrier function of retinal pigment epithelial cells, thereby promoting the occurrence and progression of uveitis (80),Therefore, modulating the activity of the IL-23/IL-17 immunological axis may offer a potential therapeutic strategy for the treatment of SpA-associated uveitis.

## 5 Advancements in treatment strategies

The choice of initial treatment for uveitis depends on many factors, including the degree of inflammation, laterality, anatomical location (anterior, middle, posterior uveitis), and the presence and extent of systemic disease (81). Current treatment strategies generally fall into two categories: local treatment and systemic treatment. A stepwise approach is typically employed, starting with less intensive measures and escalating to more powerful interventions as needed. The primary goals are to control inflammation, prevent recurrence, and restore or preserve vision.

### 5.1 Conventional treatment plan

The first-line treatment for most acute attacks of uveitis typically involves the local use of corticosteroid eye drops or ointments combined with dilators. These treatments rapidly control inflammation of the ocular surface and anterior chamber, reduce ciliary muscle spasm, prevent the formation of posterior adhesions, and offer a cost-effective therapeutic profile. At the beginning of treatment, patients are advised to apply the eye drops frequently (every half an hour to an hour), then gradually reduce the frequency of eye drops to 3–5 times a day for 1–2 weeks. The dosage is then tapered slowly to avoid the “rebound phenomenon” caused by stopping treatment too quickly (81). When choosing local eye drops, consideration should be given to their anti-inflammatory efficacy and permeability in the anterior chamber (82). If the control of anterior chamber inflammation is poor or if the uveitis involves the middle, posterior, or the entire eye, additional interventions may be necessary. These include periocular (lateral or subconjunctival) or intravitreal corticosteroid injections (83), which can supplement eye drop treatment. Studies indicate that intravitreal injection of corticosteroids are effectively against uveitic macular edema (84).

Systemic treatment includes hormone, nonsteroidal anti-inflammatory drugs (NSAIDs), disease-modifying antirheumatic drugs (DMARDs) and immunosuppressant therapy. When local treatment is ineffective or in case of refractory, chronic, posterior, and/or bilateral uveitis, systemic corticosteroid therapy is required. Treatment typically starts at an oral dose of 1 mg/kg per day and gradually decreasing over a course of 6–12 weeks (85). Although effective against both ocular and axial/peripheral joint inflammation, the long-term use of systemic corticosteroids can lead to adverse effects such as elevated blood sugar, systemic hypertension, decreased bone density, depression, and increased body weight, which limit their prolonged use (86). To mitigate these risks, the current recommended approach is to prioritize corticosteroids for initial inflammation control and transition to alternative treatment methods, such as immunosuppressants or biologics, to minimize long-term corticosteroid exposure (87).

NSAIDs are effective in controlling inflammation and are commonly used to manage postoperative inflammation and macular edema following cataract surgery (88). A study by Fiorelli et al. (89)indicated that oral NSAIDs may serve as a therapeutic option for patients with recurrent acute anterior uveitis, offering a potential transitional strategy between local therapy and long-term immunosuppression. However, the definitive efficacy of this approach requires validation through more high-quality studies. Additionally, long-term use of NSAIDs carries significant risks, including an increased incidence of gastrointestinal bleeding and perforation. Given the unclear therapeutic benefits and definite safety concerns, NSAIDs are not currently recommended for the routine management of SpA-associated uveitis.

DMARDs are not first-line but can be used as steroid-sparing therapy. Among them, sulfasalazine (SSZ) is favored for its safety and cost, and is effective mainly against peripheral joint involvement in SpA. In a 3-year prospective study, SSZ significantly reduced the frequency of recurrent AAU flare-ups (90). A recent study also confirmed that SSZ significantly and sustainably reduced the recurrence rate of AAU in NIU patients over a three-year period (91). Meanwhile, adverse effects were observed in approximately 11-40% of patients receiving SSZ, primarily including nausea, vomiting, diarrhea, headache, and loss of appetite (92). The overall safety profile of SSZ is favorable, with most adverse effects being mild. However, due to the risk of rare but severe reactions, routine safety monitoring is essential. Methotrexate (MTX) and azathioprine (AZP) are not routinely recommended for SpA-associated AAU due to insufficient evidence. Although MTX reduces uveitis relapse and complications, such as cataracts, glaucoma, and macular edema (93), and AZP has been proven to be an effective steroid-sparing therapy for chronic non-inflammatory eye diseases (94), the existing studies in SpA-AAU are limited by small samples and low evidence grade. Their definitive efficacy and safety require confirmation from large-scale, randomized controlled trials.

### 5.2 Progress in targeted therapy with biologics

In recent years, biologics have received increasing attention and shown promising therapeutic prospects. By precisely targeting specific inflammatory mediators or signaling pathways, they can rapidly control inflammation, reduce uveitis relapses, and minimize the side effects associated with traditional treatments, making therapy safer and more effective. Consequently, biological agents have emerged as a vital option in the clinical management of uveitis (**Table 2**).

**Table 2:** Targeted therapeutics for spondyloarthritis-associated uveitis.

Targeted therapy		Reference	patients	Research Methods	Result	Incidence of adverse events	Limitations
TNF inhibitors	adalimumab	Suhler et al., 2021 (97)	424	Prospective open-label study	85% achieved inflammatory remission, 89% attained steroid-free remission, with significant improvement in visual acuity	396/100 PY	Lack of control group Sample size decreases over time
	Infliximab	KWon et al., 2025 (103)	34,621	Retrospective national cohort study	Less effective than ADA, but superior to ETA	NE	Retrospective design, single ethnicity
	Golimumab	Van Bentum et al., 2019 (106)	93	Prospective historical comparison	AAU incidence rate: decreased by 80%	NE	No control group, small sample size
	Certolizumab pegol	van der Horst-Bruinsma et al., 2021 (108)	115	Prospective open-label study	AAU incidence rate: decreased by 82%	73.0%, No deaths	No control group; all subjects were HLA-B27 positive.
IL-17A inhibitors	Secukinumab	Brandt-Jürgens et al., 2025 (111)	o axSpA:1980 PsA:2453 o	*post hoc* analysis	1.29/100 PY	NE	Preliminary findings, short observation period
	Bimekizumab	Brown et al., 2024 (112)	848	Randomized double-blind extension study	The incidence of AAU in the BKZ group was significantly lower than that in the placebo group (1.8 vs 15.4 per 100 patient-years).	1.2/100PY	Lack of long-term placebo-controlled groups, self-reported
Janus kinase inhibitors	Tofacitinib	Paley et al., 2019 (116)	2	Case Report	Both cases were effective	NE	Extremely few cases, non-clinical trials
	Filgotinib	Srivastava et al., 2024 (118)	74 (Planned: 248, terminated early)	Randomized controlled trial	The treatment failure rate was significantly lower than that of the placebo (37.5% vs 67.6%)	81.1% vs 68.6%	Small sample size, early termination

PY: person-years; NA: not available; AAU: anterior uveitis; BKZ: Bimekizumab; ADA: adalimumab; ETA: etanercept.

As discussed earlier in the pathogenesis section, the pro-inflammatory cytokine TNF-α is a key factor in the development of uveitis, and its levels are upregulated in both the patient’s aqueous humor and serum. Numerous studies have confirmed that tumor necrosis factor inhibitors (TNFi) have significant therapeutic effects in treating SpA-associated uveitis. Currently, five different types of TNFi are available: monoclonal antibodies such as adalimumab (ADA), Infliximab (IFX), Golimumab (GOL), and Certolizumab pegol (CZP), as well as fusion protein etanercept (ETA).

While these agents are often used off-label for the treatment of AAU, ADA is currently the only TNFi approved for the treatment of non-infectious intermediate, posterior, or panuveitis. The VISUAL I trial included 217 participants with active non-infectious intermediate, posterior, or panuveitis. The findings demonstrated that adalimumab (40 mg administered biweekly following a loading dose) significantly decreased the risk of visual impairment in comparison to placebo (95). The subsequent VISUAL II trial in patients with quiescent, steroid-dependent disease showed a significantly lower treatment failure rate with ADA (39%) than with placebo (55%) (96). These findings were further corroborated by a subsequent multicenter study, which confirmed that long-term treatment with ADA promotes and sustains disease quiescence while also reducing dependence on systemic corticosteroids (97). Additionally, a study by Rudwaleit et al. (98) showed that ADA can reduce the attacks of SpA-associated uveitis by 51%. A meta-analysis supports that ADA can decrease the frequency of uveitis attacks, reduce recurrences, and lower the use of corticosteroids (99). A subsequent meta-analysis by Li et al. (100) further confirmed the efficacy and safety of ADA. Therefore, ADA is recommended for patients with NIU who are corticosteroid-dependent or have an inadequate response to conventional therapy. Recent studies also recommend routine ADA monitoring during adalimumab treatment to enable the maintenance of consistent therapeutic drug levels (101).

Liu et al. (102) reported that INF and ADA have similar effects in terms of therapeutic efficacy and corticosteroid-sparing. A recent retrospective nationwide cohort study by Kwon et al. (103) indicated that ADA and IFX are comparable, both effectively reducing the recurrence and incidence of AAU, and both are significantly superior to ETA. In contrast, ETA has even been reported to be ineffective and potentially associated with an increased risk of uveitis flares in some cases (104). Therefore, for patients with SpA-associated recurrent, refractory uveitis, or those for whom conventional treatment has failed, ADA or IFX are recommended as the preferred choice, while ETA should be avoided (105).

Results from a prospective study by Van Bentum et al. (106)demonstrated that GOL significantly reduced the incidence of NIU in AS patients. Other studies have also suggested that GOL is effective for refractory NIU (107). The VIEW study demonstrated that treatment with certolizumab pegol significantly reduced the incidence of AAU flares in patients with axial spondyloarthritis who have a history of AAU, suggesting it as a viable treatment option (108). However, robust evidence supporting the comparative efficacy and safety of GOL and CZP is still lacking, and further studies are needed to provide a more substantial evidence base. In summary, the role of TNFi in managing SpA-associated uveitis is considerable. Nevertheless, in clinical practice, serious attention must be paid to their potential side effects and adverse reactions.

IL-17A inhibitors (IL-17Ai) work by targeting IL-17A to reduce inflammation and improve clinical symptoms. However, their role in preventing or reducing AAU recurrence is not yet clear. Secukinumab, a widely used IL-17Ai, has been trialed for its efficacy in uveitis. A nationwide observational cohort study of SpA patients suggested that those using secukinumab may have higher risk of AAU compared with monoclonal TNFi (109). Additionally, the results of three randomized controlled trials suggested that secukinumab failed to meet its primary efficacy endpoints in the treatment of non-infectious uveitis, indicating that its efficacy in this area may be limited (110). In a *post-hoc* analysis of 11 trials, secukinumab demonstrated a numerically lower AAU incidence than placebo in axSpA (1.29 vs. 1.72), though not significant (111). In summary, IL-17A inhibitors play a limited role in preventing spondyloarthritis-associated uveitis and are less effective than TNFi. Consequently, they are not recommended as the first-choice biologic treatment for patients with a history of recurrent AAU. Bimekizumab (BKZ), a dual IL-17A/F inhibitor, has been shown to significantly reduce the rate of uveitis recurrence, indicating its potential therapeutic value (112). However, its efficacy and safety necessitate further validation.

Targeting the IL-12/IL-23 pathway with biologic agents is another potential therapeutic strategy for patients who experience failure or intolerance to TNFi and/or IL-17A inhibitors (18). Ustekinumab is a monoclonal antibody that blocks the IL-12/IL-23 pathway, primarily used in the treatment of autoimmune diseases such as psoriatic arthritis and Crohn’s disease. Studies have demonstrated its therapeutic efficacy on SpA-associated uveitis, such as psoriatic arthritis related uveitis (113) and inflammatory bowel disease-related uveitis (114). However, these conclusions are predominantly based on small-sample or retrospective studies and lack validation from randomized controlled trials. Therefore, the therapeutic role of ustekinumab in SpA-associated uveitis requires confirmation through more high-quality studies.

Janus kinase inhibitors (JAKi) inhibit intracellular signaling by blocking the JAK pathway, thereby reducing the production of inflammatory cytokines and effectively mitigating the inflammatory response. Tofacitinib is a representative drug of this class, selectively inhibiting the activity of JAK1 and JAK3 (115). It has been reported that tofacitinib is effective in controlling refractory ocular inflammation after failed steroid-sparing treatment (116). Studies have shown that tofacitinib can also treat juvenile idiopathic arthritis-related uveitis without significant side effects (117). The phase 2 HUMBOLDT trial (118) found filgotinib significantly reduced treatment failure by 30.1% vs placebo in active non-infectious uveitis, though adverse events were higher. Furthermore, compared to TNFi or IL-17Ai, the clinical cohort for JAKi in SpA-associated uveitis remains small, with limited long-term safety data. Thus, further evaluation through more extensive studies is urgently needed. As clinical trials progress and treatment experience accumulates, JAKi hold promise as a novel therapeutic strategy for the management of SpA-associated uveitis.

To achieve optimal management, ophthalmologists and rheumatologists need to collaborate closely in key areas such as diagnosis, early referral, decision-making, and follow-up care. Additionally, the personalization of treatment plans is crucial, with strategies tailored to the specific conditions of each patient. Through this individualized and comprehensive treatment approach, the negative impact of uveitis on patients’ vision and quality of life can be minimized to the greatest extent.

## 6 Summary and outlook

SpA-associated uveitis is a significant extra-articular manifestation that greatly impact the visual health of patients. Early diagnosis and timely treatment are crucial to prevent serious complications and protect vision. Detailed ophthalmic examinations and comprehensive systemic assessments should be conducted for SpA patients, and vigilance should be maintained regarding the potential impact of associated risk factors. Future research should focus on further understanding the pathogenesis of SpA-associated uveitis in order to develop more effective prevention and treatment strategies. While most patients have a good response to local corticosteroid therapy, recurrent or refractory patients often require a more comprehensive treatment approach, including systemic corticosteroids, NSAIDs, DMARDS, or biologics.

Biological agents, especially those targeting specific inflammatory pathways, have shown unique advantages in long-term control of joint and ocular inflammation. However, their efficacy and safety need to be further validated through extensive research. JAK inhibitors, as an emerging treatment option, provide a new treatment approach for SpA-associated uveitis. Although their efficacy and safety remain under investigation, preliminary studies highlight their potential in regulating immune responses and reducing inflammation.

In this review, we discussed in detail the clinical characteristics and popular hypothesis for the pathogenesis of SpA-associated uveitis, as well as provided an overview of current treatment approaches, including novel treatment strategies targeting immune components. Effective management of SpA-associated uveitis requires close collaboration between ophthalmologists and rheumatologists, providing patients with personalized and comprehensive treatment strategies through multidisciplinary teams. This collaborative approach not only improve treatment effectiveness, but also reduce risk of missed or incorrect diagnosis, thereby minimizing the impact of the disease on patients’ vision and quality of life.
